# Posteromedial capsular anatomy of the tibia for consideration of the medial meniscal support structure using a multidimensional analysis

**DOI:** 10.1038/s41598-023-38994-x

**Published:** 2023-07-25

**Authors:** Masahiro Tsutsumi, Akimoto Nimura, Suthasinee Tharnmanularp, Shintarou Kudo, Keiichi Akita

**Affiliations:** 1 Inclusive Medical Sciences Research Institute, Morinomiya University of Medical Sciences, 1-26-16 Nankokita, Suminoe-ku, Osaka City, Osaka 559-8611 Japan; 2 Department of Clinical Anatomy, Graduate School of Medical and Dental Sciences, Tokyo Medical and Dental University, Tokyo, Japan; 3 Department of Functional Joint Anatomy, Graduate School of Medical and Dental Sciences, Tokyo Medical and Dental University, Tokyo, Japan

**Keywords:** Musculoskeletal system, Orthopaedics, Rehabilitation

## Abstract

Medial meniscal extrusion (MME) is a structural abnormality that leads to early knee osteoarthritis; however, its formation remains debated. For anatomical consideration of the mechanism underlying MME formation, we examined the capsular attachment on the posteromedial tibia and its layered association with the semimembranosus. Fourteen knees of eight body donors were analyzed in this study; six knees were grouped for macroscopic analysis, whereas four knees each were grouped for histological and phosphotungstic acid-enhanced micro-computed tomography analyses. The capsular attachment varied in width according to location and was not distant from the articular cartilage and posterior root. A portion of the posteromedial joint capsule formed the semimembranosus tendinous sheath. The dense fibrous membrane superficial to the semimembranosus, which was continuous from its tendinous sheath, existed as one of the layers of the joint capsule. The aforementioned findings were confirmed in all specimens. Based on the capsular attachment and its layered association with the semimembranosus, the conventional posteromedial knee ligaments may be only a part of the joint capsule divided into two layers by the semimembranosus. If the coordinated action of the joint capsule and semimembranosus partially contributes to the medial meniscus stability, such a structural problem may affect MME formation.

## Introduction

It is challenging to elucidate the mechanisms for initiation/progression of early knee osteoarthritis in order to establish prevention methods. Medial meniscal extrusion (MME) is the displacement of the medial meniscus from the tibial articular cartilage or the uncovering of the tibial articular cartilage by the medial meniscus; it is one of the structural abnormalities believed to be associated with the initiation and progression of knee osteoarthritis^1–3^. However, the origins and pathophysiology of MME formation itself remain debated. Previous studies have reported that posterior root tears of the medial meniscus^4–6^ and osteophyte formation at the medial tibia^7,8^ contribute to MME formation. However, these conditions may have occurred after prolonged degeneration^9^ or repetitive stress^10,11^; therefore, it is preferable to consider other mechanisms of pathological initiation that can be targeted for early prevention of MME. Moreover, to gain a more comprehensive picture of MME formation, precise anatomical knowledge regarding medial meniscus stabilization may be essential.

**Figure 1:**
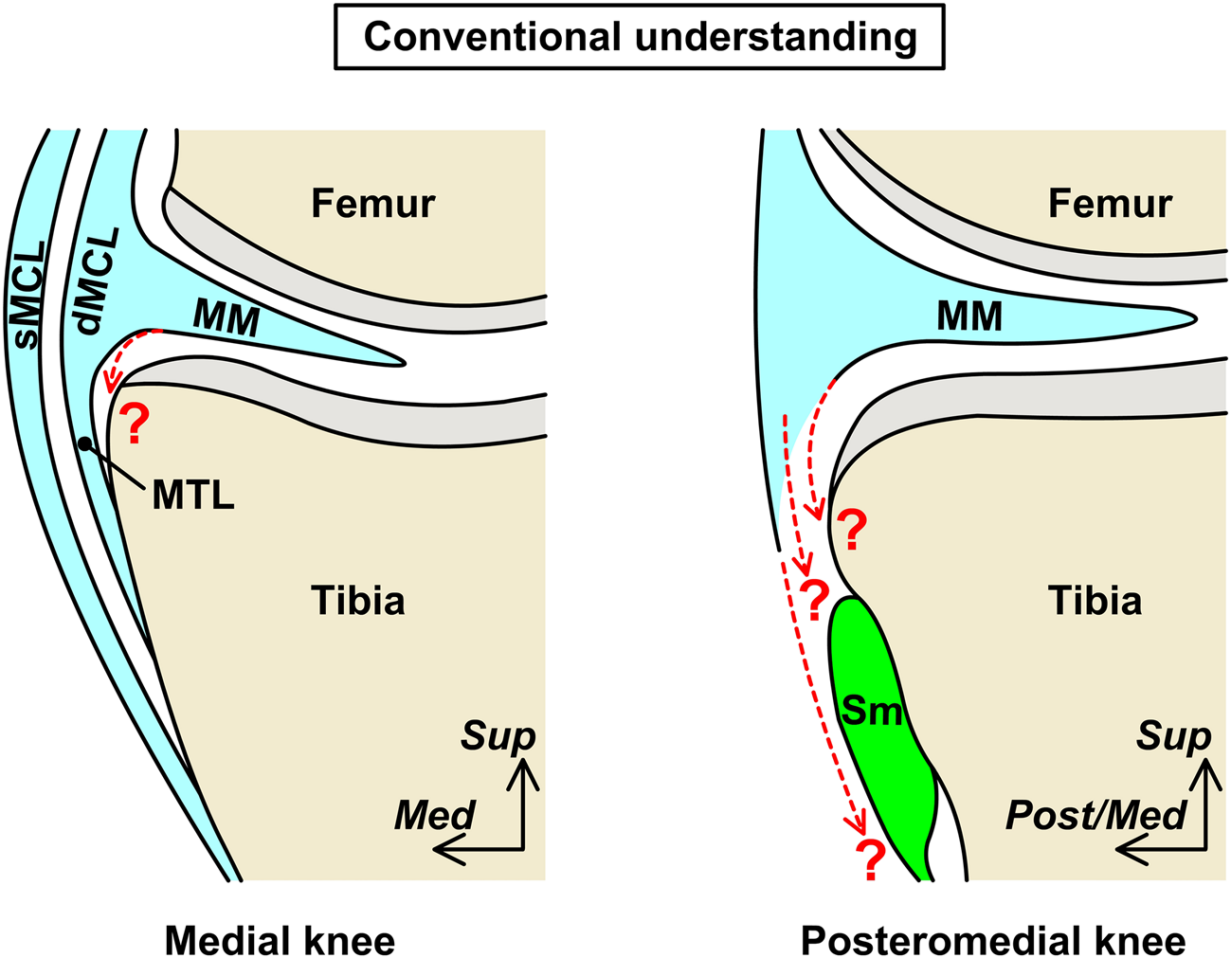
Conventional understanding of the support structures for the medial meniscus at the medial and posteromedial knees. Schematic illustration of the cross sections at the medial (left panel) and posteromedial (right panel) knees. The superficial and deep medial collateral ligaments (sMCL and dMCL, respectively) form two layers on the medial side of the knee, and the medial meniscus (MM) attaches to the tibia via the meniscotibial ligament (MTL). However, whether the joint capsule is located at a separate layer from these two layers and where the joint capsule attaches are not well understood. Additionally, where the semimembranosus (Sm) locates against these two layers on the posteromedial side of the knee, and whether it is more superficial or deep than the joint capsule is unknown. *Med* Medial, *Post/Med* Posteromedial, *Sup* Superior.

There are two types of joint stabilization mechanisms: static (provided by the surrounding ligaments) and dynamic (provided by the adjacent muscles). Regarding the static stabilizer of the medial meniscus, studies have focused on the posteromedial ligaments and their tibial attachment, including the superficial medial collateral ligament (sMCL; also known as the tibial collateral ligament), deep medial collateral ligament (dMCL), posterior oblique ligament (POL), and meniscotibial (coronary) ligament (MTL)^12–17^. Regarding the dynamic stabilizer, most studies have concentrated on the semimembranosus^15,18,19^. However, the capsular attachment on the posteromedial tibia has rarely been investigated despite the medial meniscus forming a complex with the joint capsule^12,20^. In addition, the layered structure in the joint capsule and pericapsular muscles is useful in understanding the interrelationships between the static and dynamic stabilizers in most joints^21^, including the knee^22,23^. The semimembranosus can be regarded as the knee pericapsular muscle and has been well discussed regarding its connection to the joint capsule^15,18,19^. However, their layered relationships remain unclear due to the lack of histological analysis, which has only been analyzed macroscopically^14^. For example, while the sMCL and dMCL form two layers on the medial side of the knee^12,14–16^, whether the joint capsule forms a separate layer from these layers is unknown. Furthermore, where the semimembranosus locates against these two layers on the posteromedial side of the knee and whether it is more superficial or deep than the joint capsule is unclear (Fig. 1). It is difficult to observe uninterrupted continuous sections during histological analysis; however, this limitation can be compensated by phosphotungstic acid-enhanced micro-computed tomography (micro-CT) analysis, which can obtain any sectional information of the soft tissue^24–28^.

This study aimed to investigate the capsular attachment on the posteromedial tibia and its layered association with the semimembranosus using an integrated multidimensional analysis incorporating macroscopic, histological, and micro-CT analyses. We hypothesized that the capsular attachment on the posteromedial tibia varied according to location and the layered relationships between the semimembranosus and joint capsule might suggest the presence of interrelationships between the static and dynamic stabilizers of the medial meniscus.

## Results

### Macroscopic analysis showing semimembranosus and posteromedial capsular attachment on the tibia

**Figure 2:**
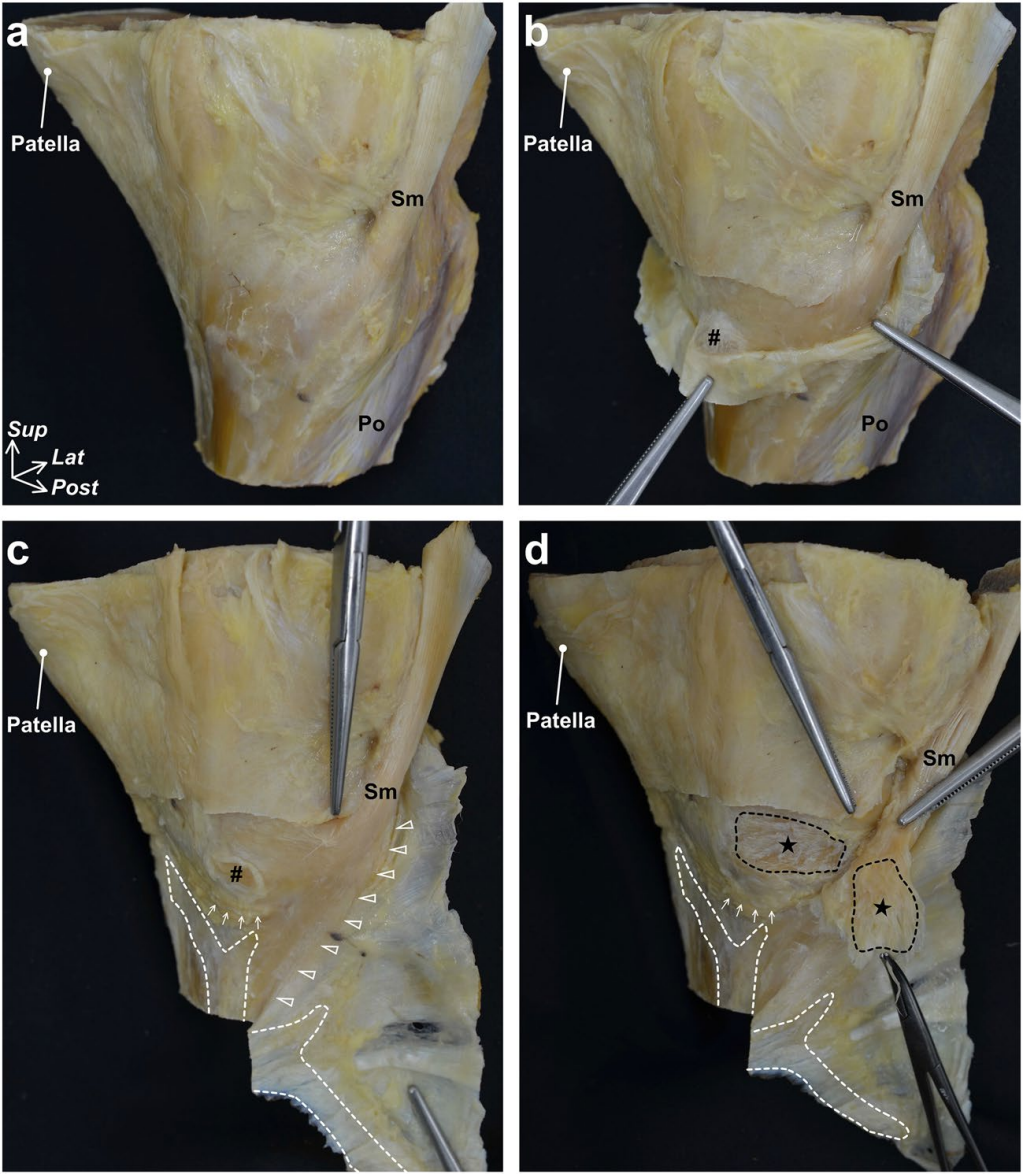
Semimembranosus attachment on the superior edge of the tibia and its tendinous sheath. The posteromedial aspect of the right knee. (**a**) The distal tendon of the semimembranosus (Sm) is covered with its tendinous sheath. (**b**) After cutting through the superior margin of the tendinous sheath, the distal Sm tendon is partially exposed. The tendinous sheath comprises a part of the joint capsule and continues to the tendinous membrane anterior to the Sm tendon. The synovial bursa (sharp) is located between the anterior edge of the Sm and the tendinous membrane. (**c**) By detaching the tibial attachment of the tendinous membrane (white dotted line), it becomes continuous with the posterior portion of the Sm (arrowheads). (**d**) The Sm partially attaches on the superior edge of the tibia (star), and this attachment is located superior to the inferior medial genicular artery (arrows). *Po* Popliteus, calcaneus, *Lat* Lateral, *Post* Posterior, *Sup* Superior.

On the posteromedial aspect of the knee, the insertion tendon of the semimembranosus was covered with a tendinous sheath (Fig. 2a). This tendinous sheath comprised a part of the joint capsule and continued to the tendinous membrane, which is located anterior to the semimembranosus tendon (Fig. 2b). In the superficial layer of the semimembranosus, cord-like structures completely independent of the tendinous membrane were not observed. The tendinous membrane and anterior edge of the semimembranosus were separated from each other by the synovial bursa but were partly connected to the posterior portion of the semimembranosus (Fig. 2c). The semimembranosus was attached to the superior edge of the posteromedial tibia with a distinct bony impression (Fig. 2d and Supplementary Fig. S1). The above findings (Fig. 2a–d) were observed in all six knees examined macroscopically (i.e., in all specimens).

**Figure 3:**
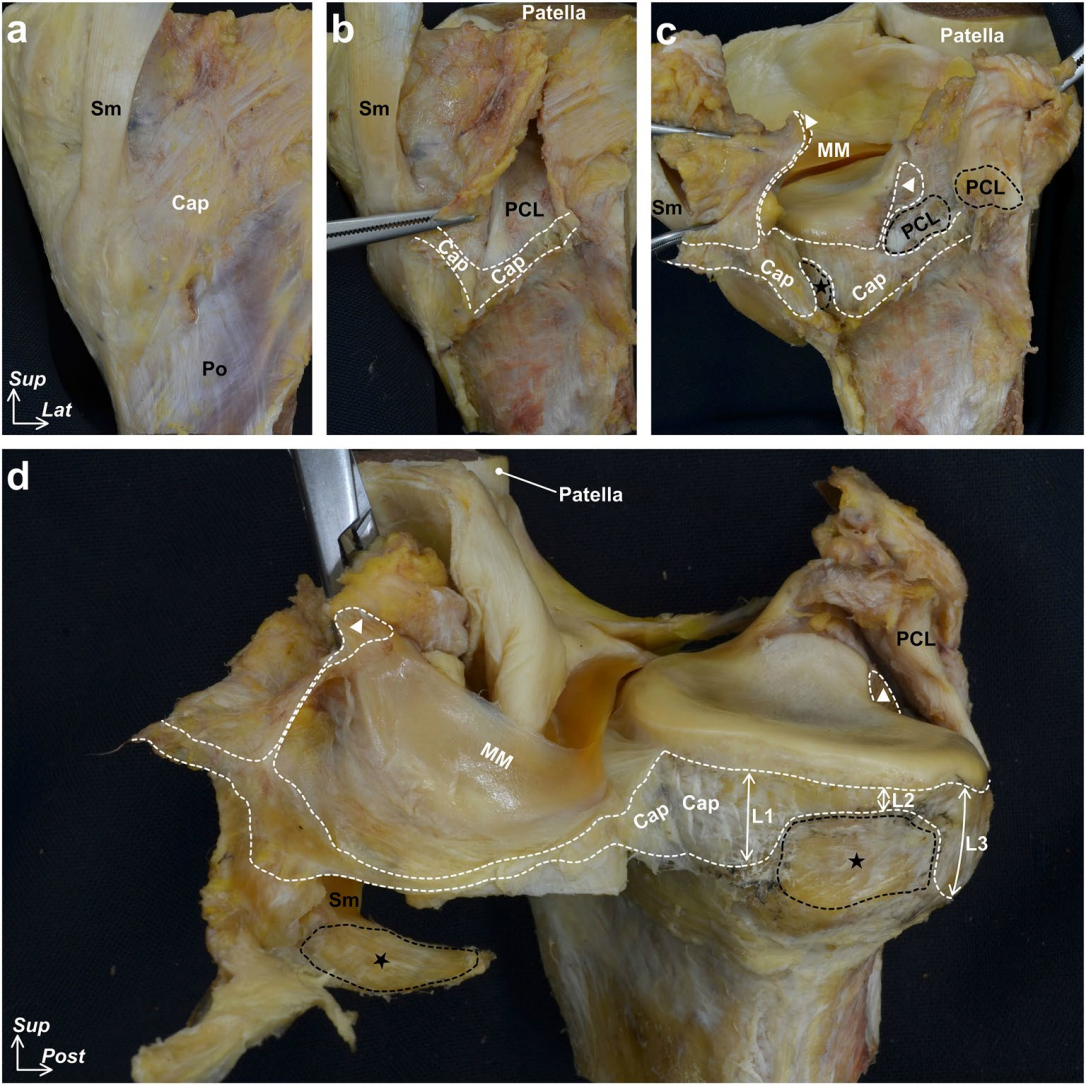
Posteromedial capsular attachment on the superior edge of the tibia. (**a**) Posterior aspect of the right knee. The semimembranosus (Sm) tendon is continuous with the posterior joint capsule (Cap) and superficial tendinous membrane of the popliteus (Po). (**b**) After removal of the popliteus, the Cap complex with the Sm is detached from the superior edge of the tibia (white dotted line). (**c**) After detaching the posterior cruciate ligament (PCL), the posterior horn of the medial meniscus (MM) is detached from the tibia to detach the Cap more medially. The posterior root area (triangle) is continuous with the posterior Cap attachment area. (**d**) Medial aspect of the knee. After detaching the Cap anteriorly to the Sm attachment (star), its attachment widths were measured at the anterior and posterior edges of the Sm attachment (L1 and L3), and the minimum width was measured superior to the Sm attachment (L2). *Lat* Lateral, *Post* Posterior, *Sup* Superior.

On the posterior aspect of the knee, the semimembranosus tendon was also continuous with the posterior joint capsule (Fig. 3a). The joint capsule was attached to the posteromedial tibia with widths that varied according to location. The medial meniscus was continuous with the joint capsule over the entire circumference, and its posterior root area, which is anterior to the posterior cruciate ligament, was also not independent of the capsular attachment area on the tibia (Fig. 3b,c). The above findings were also observed in all six specimens.

The widths of capsular attachment significantly varied according to location [one-way analysis of variance (ANOVA), *p* < 0.001; anterior to the semimembranosus attachment (Fig. 3d, L1 = 15.1 ± 1.5 mm) vs. superior to the semimembranosus attachment (Fig. 3d, L2 = 5.4 ± 1.7 mm), *p* < 0.001; L2 vs. posterior to the semimembranosus attachment (Fig. 3d, L3 = 12.6 ± 2.0 mm), *p* < 0.001]. The width of the capsular attachment was relatively wide posterior to the semimembranosus attachment on the superior edge of the tibia, decreased superior to the semimembranosus attachment, and increased anterior to the semimembranosus attachment in all specimens (Fig. 3d). The width measurements are summarized in Table 1.

**Table 1:** Width of the capsular attachments.

Measurement location	Width (mm)
Anterior edges of the Sm attachment (L1)	15.1 ± 1.5*
Minimum width superior to the Sm attachment (L2)	5.4 ± 1.7
Posterior edges of the Sm attachment (L3)	12.6 ± 2.0*

Measurement locations are shown in Fig. 3d. The width is expressed as mean and standard deviation.

*Sm* Semimembranosus.

*p < 0.001 as compared with L2.

### Histological and micro-CT analyses showing the layered relationships between the semimembranosus and posteromedial joint capsule

**Figure 4:**
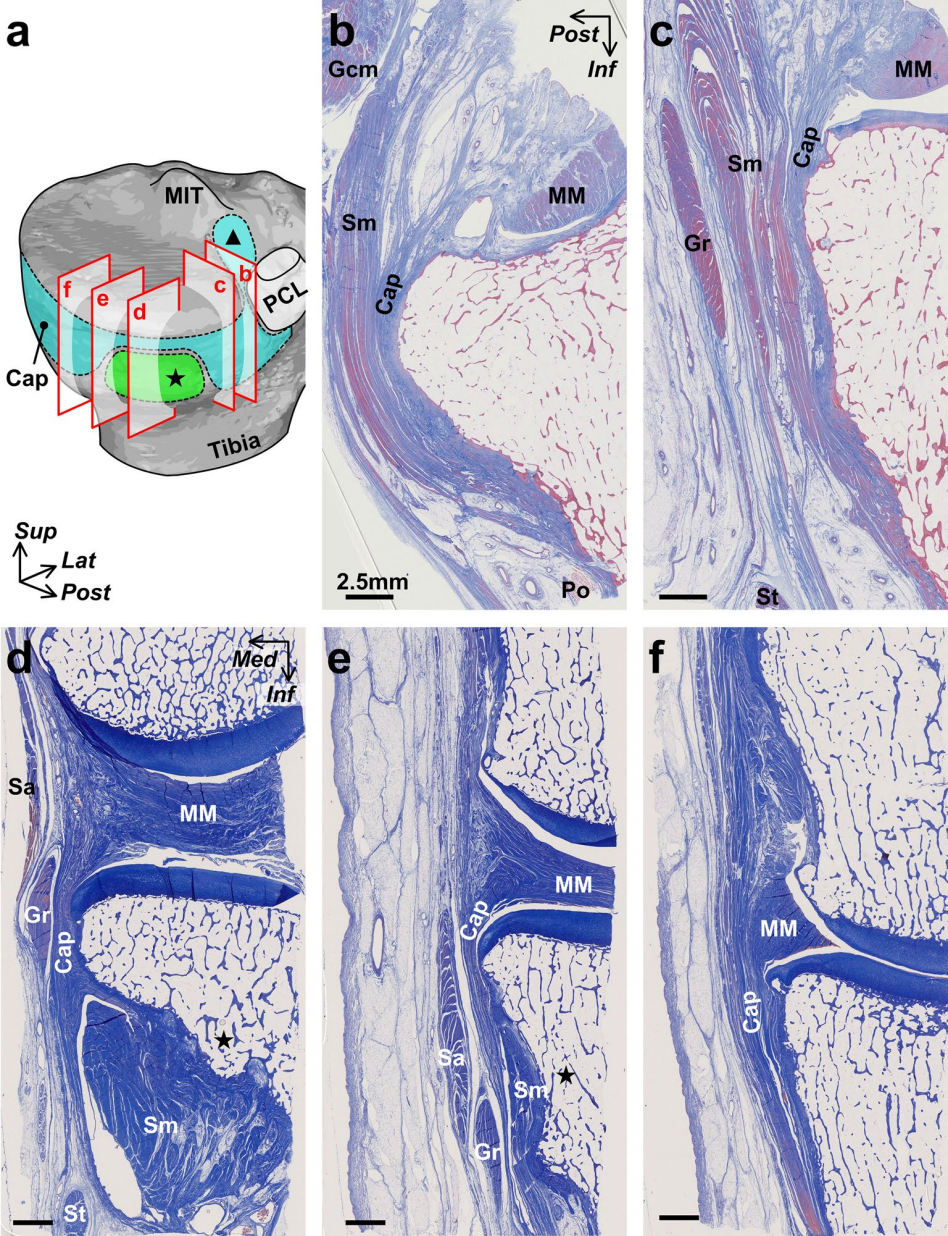
Histological analysis of the joint capsule and semimembranosus (Masson’s trichrome staining). Schematic illustration (**a**), the posteromedial aspect of the right medial knee, indicates histological section locations in sagittal (**b** and** c**) and coronal sections (**d**–**f**). The joint capsule (Cap) is not histologically independent from the medial meniscus (MM) and attaches to the superior edge of the tibia without any distance from the articular cartilage. The semimembranosus (Sm) is in close contact with the posterior Cap (**b**); it is gradually covered by a loose tendinous sheath at the posteromedial region (**c**) and completely covered by a dense fibrous sheath continuous with the Cap at the proximal tibial attachment (Star, **d**, **e**). Scale bars = 2.5 mm. *Gcm* Medial head of the gastrocnemius, *Gr* Gracilis, *MIT* Medial intercondylar tubercle, *PCL* Posterior cruciate ligament, *Po* Popliteus, *Sa* Sartorius, *St* Semitendinosus, *Triangle* Posterior root area of the MM, *Inf* Inferior, *Med* Medial, *Lat* Lateral, *Post* Posterior, *Sup* Superior.

**Figure 5:**
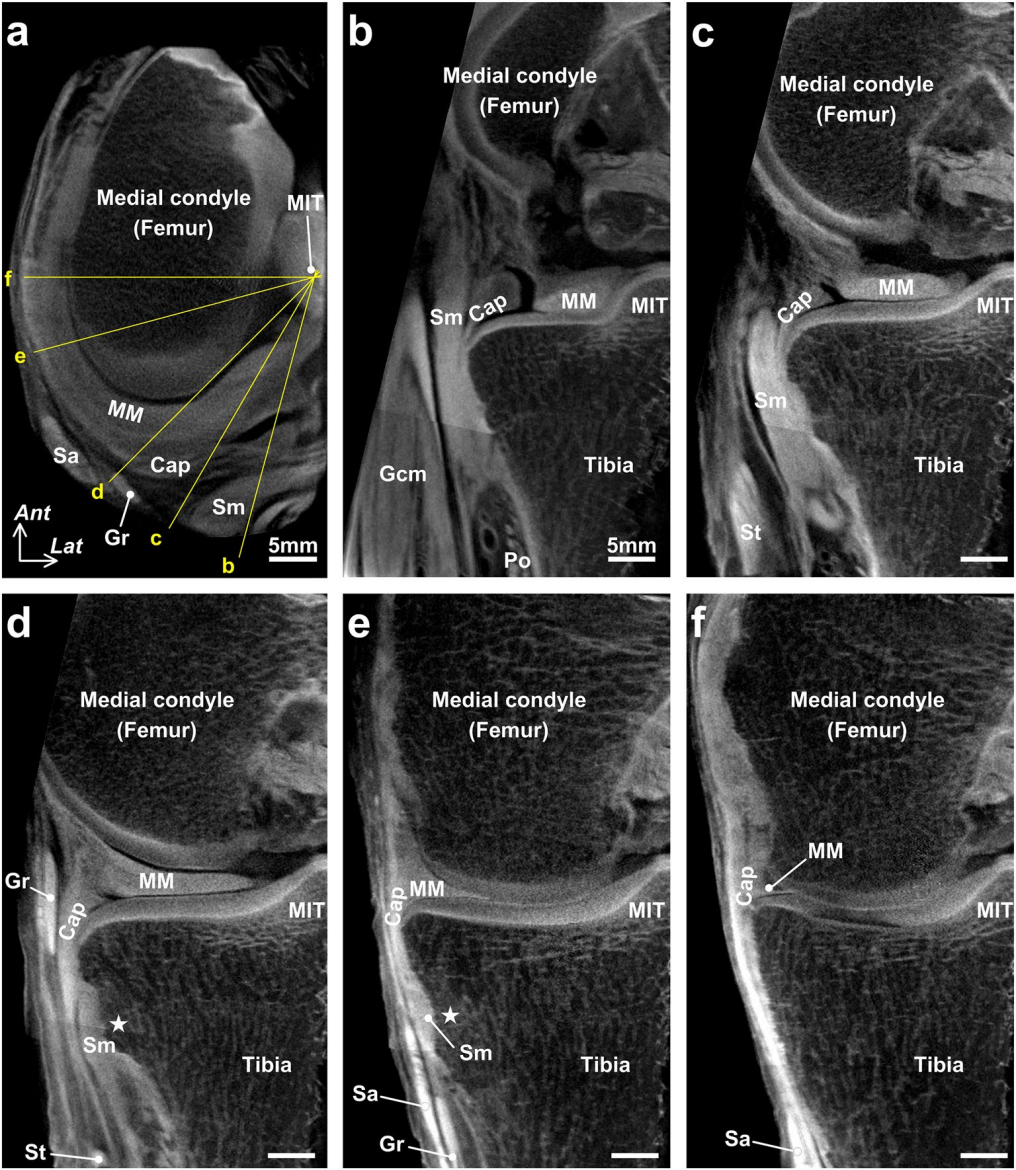
Serial radial sections of the joint capsule and semimembranosus using phosphotungstic acid-enhanced micro-CT. Horizontal enhanced micro-CT image using phosphotungstic acid (**a**) indicates the radial slice locations centered around the medial intercondylar tubercle (MIT) for visualizing the following sections: images with 15° (**b**), 30° (**c**), 45° (**d**), 75° (**e**), 90° (**f**) clockwise from sagittal sections. Scale bars = 5 mm. *Cap* Joint capsule, *Gcm* Medial head of the gastrocnemius, *Gr* Gracilis, *MM* Medial meniscus, *Po* Popliteus, *Sa* Sartorius, *Sm* Semimembranosus, *St* Semitendinosus, *Star* Sm attachment on the superior edge of the tibia, *Ant* Anterior, *Lat* Lateral.

The joint capsule was confirmed to be not histologically independent of the medial meniscus over the entire circumference (Fig. 4a–f). The joint capsule was attached to the posteromedial tibia via fibrocartilage, even where the width of the capsular attachment was narrow (Fig. 4d,e). In addition, the upper margin of the capsular attachment reached the lower margin of the articular cartilage without a gap in any histological section. The semimembranosus was in close contact with the posterior joint capsule (Fig. 4b) and was gradually covered by a loose tendinous sheath at the posteromedial region (Fig. 4c). Furthermore, it was completely covered by a dense fibrous sheath, which was continuous with the posteromedial joint capsule from the posterior to the anterior edge of the proximal tibial attachment (Fig. 4d,e). At the medial side of the tibia anterior to the semimembranosus attachment, the dense fibrous membrane, which was also continuous with the joint capsule, occupied the same layer as the dense fibrous sheath of the semimembranosus (Fig. 4f). The above-mentioned histological findings were based on sagittal and coronal sections of different specimens; however, the radial sequential images obtained from the micro-CT analysis confirmed the layered characteristics from the posterior to medial knee in the same specimen (Fig. 5 and Supplementary Video S1).

## Discussion

**Figure 6:**
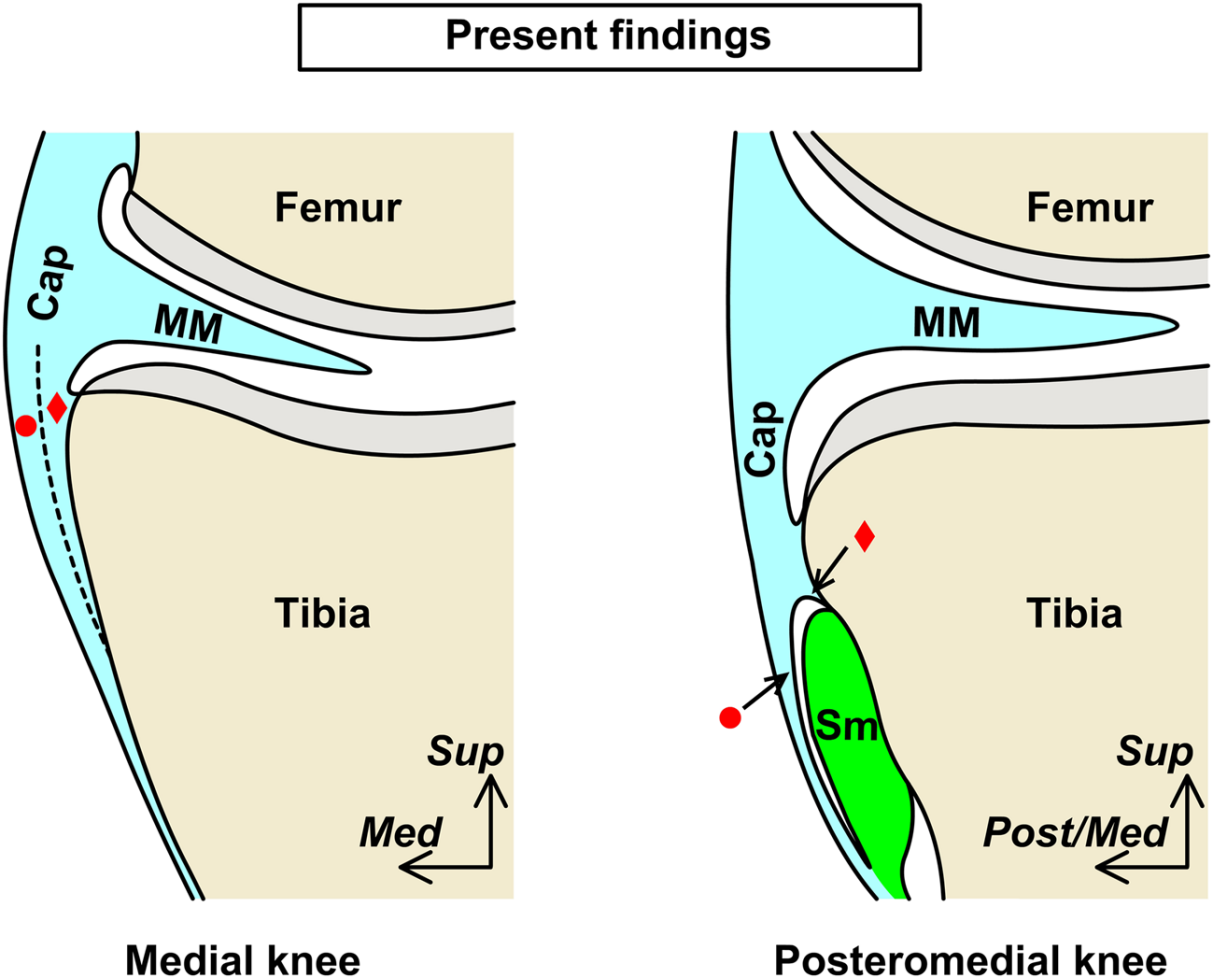
The layered structure of the joint capsule and semimembranosus. Schematic illustration of the cross sections at the medial (left panel) and posteromedial (right panel) knees. The medial meniscus (MM) attached to the tibia via the joint capsule (Cap), with the attachment varying in width according to location and not distant from the articular cartilage. A part of the posteromedial Cap forms the tendinous sheath of the semimembranosus (Sm). The fibrous membrane superficial to the Sm, which is continuous from the Sm tendinous sheath, exists as one of the layers of the Cap (indicated by red circles). The red diamonds also indicate the corresponding layer of the Cap. *Med* Medial, *Post/Med* Posteromedial, *Sup* Superior.

The present study revealed that the capsular attachment width was wide posterior and anterior to the semimembranosus attachment on the superior edge of the tibia and narrow superior to the semimembranosus attachment. This attachment was not independent of the posterior root of the medial meniscus and was histologically close to the articular cartilage. A portion of the posteromedial joint capsule formed the semimembranosus tendinous sheath. This tendinous sheath gradually became dense fibrous tissue from the posterior to the medial and continued to the dense fibrous membrane as one of the layers of the joint capsule. The medial meniscus was attached to the tibia through the joint capsule and was continuous with the joint capsule over the entire circumference. Our main findings were summarized in Fig. 6.

The capsular attachment in the present study varied in width according to location (range, approximately 5–15 mm) and was not distant from the articular cartilage and posterior root. In a previous report regarding capsular attachment, Warren and Marshall reported, “*its lines of attachment above and below the joint are essentially the same as the margins of the articular surfaces*”^14^. Therefore, our results were consistent with the previous study regarding distance to the articular cartilage but differ in terms of the attachment width, which was not linear, and focusing on the proximity to the posterior root. This discrepancy may be attributed to the conventional viewpoint that only the synovial membrane, which constitutes the joint capsule, has been considered the joint capsule, while the fibrous membrane of the joint capsule, is regarded as the MTL. Thus, the general emphasis is that the medial meniscus attaches to the tibia through the MTL rather than the joint capsule^20^. Additionally, the width of the MTL attachment has been considered to vary between 9 and 17 mm according to location^18^ and was approximately the same as that of the capsular attachment reported in this study. Because the synovial membrane closely lines the fibrous membrane in most articular systems^29^, it is improbable that synovial and fibrous membranes could independently move during knee movement. Therefore, it is anatomically and possibly functionally accurate to interpret the medial meniscus as attached to the tibia via the joint capsule, with the capsular attachment being continuous with the posterior root, rather than assuming that the medial meniscus attaches to the tibia via the MTL.

We showed that a dense fibrous membrane continuous from the semimembranosus tendinous sheath was a layer of the joint capsule. Warren and Marshall^14^ reported the sMCL as superficial to the semimembranosus instead of identifying this fibrous membrane reported in our study. Regarding this fibrous membrane, previous studies have reported similar structures continuous with the sMCL^12,30^. Furthermore, many studies have shown that the sMCL can be separated from the joint capsule^12,14–16^; however, these studies only pointed out the differences in the layers, with none reporting the clear anterior and posterior boundaries with surrounding structures. Therefore, on the basis of the observed layered relationships, the sMCL may be interpreted as a part of the dense fibrous membrane that is continuous from the semimembranosus tendon sheath rather than a distinct cord-like structure.

Distinct cord-like structures like the dMCL in the same layer as the capsule^14^ and those like the POL surrounding the semimembranosus^13,15^ were not observed in the present study, unlike that in previous studies. As most studies have pointed out, the dMCL is interpreted as a constituent of the joint capsule^14–16^; the POL is merely a subdivision, based on its proximal attachment, of what was originally considered the sMCL^13,15^. Therefore, the “posteromedial knee ligaments” may be nothing more than a regionally named structure that is a joint capsule divided into two layers by the tendon.

Regarding clinical relevance, the present study provided two hypothetical insights regarding MME formation. First, the present study suggested that the medial meniscus may be cooperatively stabilized by the posterior root and joint capsule based on the continuity between the posterior root and capsular attachment. A recent meta-analysis and systematic review suggested that surgical repair of the posterior root biomechanically improved knee contact pressure but did not clinically improve the MME^31^. Therefore, the posterior root is not considered to support the meniscus independently. The peripheral capsule yielded a smaller tensile modulus than did the meniscus itself or the posterior root^32,33^; however, the peripheral capsule was suggested to have a sufficient role in maintaining the circumferential hoop tension of the meniscus^32^. Additionally, the disruption of the MTL, namely the capsular attachment on the tibia, was reported to have a biomechanically significant effect on knee stability^34,35^. According to Krych et al., the disruption of the MTL was a predisposing event that contributed to the posterior root tears of the medial meniscus^36^. Therefore, a capsular abnormality may trigger the accumulation of mechanical stress on the posterior root and its tear, although the contribution of the capsular abnormality to MME is assumed to be smaller than that of the posterior root.

Second, according to Hada et al., the medial tibial osteophyte in early knee osteoarthritis occurred close to the articular cartilage and was closely associated with the MME^7^. The present study revealed that the joint capsule was attached close to the articular cartilage where osteophytes can form; therefore, a capsular abnormality may precede osteophyte formation. Because the medial meniscus was continuous with the joint capsule, which formed the semimembranosus tendinous sheath, the joint capsule may dynamically coordinate the medial meniscus by transmitting semimembranosus action through the tendinous sheaths. Therefore, based on our interpretation that existing ligaments are part of a joint capsule, the dynamic and static stabilizers of the medial meniscus may cooperatively function. We believe structural abnormalities of the joint capsule precede the posterior root tear or osteophyte formation.

This study had some limitations. First, this study was purely anatomical and limited to uninjured specimens; therefore, we could not prove the mechanism of MME formation, and our explanations remain speculative. Since the structural abnormalities of the joint capsule might be interpreted as an early causative factor for MME, examining these abnormalities in depth will be essential in the future. For example, questions on whether MME formation is caused by an attachment abnormality, such as one involving the synovial membrane, or whether MME formation is associated with the semimembranosus should be investigated. Second, the sample size was relatively small. However, we compensated for the small sample size with a multidimensional analysis, which included macroscopic, histological, and micro-CT analyses. Third, the lack of radiographic evaluation meant we could not entirely exclude the knee with osteoarthritis despite this study aiming to analyze the normal structure. Finally, the advanced age of the individuals from whom the cadaveric specimens were obtained might have affected our findings. However, this effect may be negligible because patients with knee osteoarthritis are also often relatively old.

In conclusion, the capsular attachment on the posteromedial tibia varied in width according to location and was not distant from the posterior root. The joint capsule formed the semimembranosus tendinous sheath, and the conventional posteromedial knee ligaments may only be a part of the joint capsule divided into two layers by the semimembranosus tendon. If the coordinated action of the joint capsule and semimembranosus partially contributes to the medial meniscus stability, such a structural problem may affect MME formation.

## Methods

### Specimen preparation

Sixteen knees from ten Japanese body donors (six males and four females; mean age at death: 80.9 years) donated to the Department of Anatomy at Tokyo Medical and Dental University were used in this study. Before their death, all donors voluntarily declared that their remains be donated for education and research. This study complied with the Japanese law entitled “Act on Body Donation for Medical and Dental Education,” and the study design was approved by the Medical Research Ethics Committee of Tokyo Medical and Dental University (approval no.: #M2018-243-01). In addition, all procedures were performed in accordance with the Japanese guidelines entitled “Ethical Guidelines for Medical and Health Research Involving Human Subjects”.

The anatomic specimens without surgical history in the lower extremity were included in this study, fixed with 8% formalin, and preserved in 30% ethanol. Using a diamond saw (EXAKT 312; EXAKT Advanced Technologies, Norderstedt, Germany), the knee specimens were obtained by cutting proximal at the center of the patella and distal at the superior one-third of the tibia. Then, the medial half of the knee was acquired by cutting off the lateral half, leaving the medial meniscus undamaged. Based on the half-sectioned aspect, all specimens were macroscopically assessed for distinct intra-articular abnormalities such as osteoarthritis changes. In addition, the sectioned specimens underwent micro-CT (inspeXio SMX-100CT; Shimadzu, Kyoto, Japan) with a 200-µm resolution to determine if any bony abnormalities were present. From the series of micro-CT images, three-dimensional reconstruction images were obtained using ImageJ software version 1.53 (National Institutes of Health, Bethesda, MD, USA). Two knees of two cadavers were excluded from the analysis because of osteoarthritis changes observed during the macroscopic and micro-CT assessment of the specimens.

Therefore, 14 knees of eight body donors (five males and three females; mean age at death: 78.6 years) were finally analyzed in this study. Six knees of four body donors were randomly grouped for macroscopic analysis, whereas the remaining eight knees of four body donors were grouped for histological (one side of each donor) and phosphotungstic acid-enhanced micro-CT (the other side of each donor) analyses.

### Macroscopic analysis of the posteromedial region of the tibia

For the macroscopic analysis (six knees; three males and one female; mean age at death: 82.8 years), the skin and subcutaneous tissues, muscles comprising the pes anserinus (sartorius, gracilis, and semitendinosus), and medial head of the gastrocnemius were removed. The femur was also removed from the specimen by detaching its capsular attachment. Then, we analyzed the layered relationships and connection to the semimembranosus, joint capsule, and medial meniscus and their attachments to the posteromedial region of the tibia. In addition, the attachment width of the joint capsule on the posteromedial tibia was measured. Measurements were performed twice by a single examiner, and the average of two measurements was recorded for statistical analysis. Intraclass correlation coefficients (ICCs) were calculated to determine the intra-rater reliability of each measured value.

### Histological analysis of the posteromedial region of the knee

The four knees (two males and two females; mean age at death: 74.5 years) grouped for histological analysis were embedded in a 3% agar solution and frozen at − 80 °C. Two specimens were then serially sectioned into 5-mm thick segments in the sagittal plane using a band saw (WN-25-3; Nakajima Seisakusho, Osaka, Japan), whereas the remaining two knees were sectioned in the coronal plane. Each section level was identified based on the correspondence between the section bony morphology and that observed on the micro-CT image at the specimen preparation. After removing the excess agar, we chose two sagittal sections at the following levels: immediately medial to the posterior cruciate ligament and its adjacent section, and three coronal sections: posterior and anterior edges of the semimembranosus attachment to the superior end of the tibia and the section anterior to the semimembranosus attachment. Subsequently, we harvested a block from each section, including the semimembranosus, joint capsule, and medial meniscus; the blocks were decalcified for 3 weeks with Plank–Rychlo solution (AlCl_3_:6H_2_O [70.0 g/L], HCl [85.0 mL/L], and HCOOH [50.0 mL/L])^37^. After decalcification, each block was dehydrated and embedded in paraffin. The paraffin-embedded tissue was sliced into 5 μm sections with a 1-mm interval; the sections were stained using the Masson trichrome staining protocol.

### Analysis of the posteromedial region of the knee using phosphotungstic acid-enhanced micro-CT

Four knees were analyzed using phosphotungstic acid-enhanced micro-CT. The specimens were dehydrated in 70% ethanol solutions for a day and then stained with 1% phosphotungstic acid solution in 70% ethanol for 16–20 weeks. The stained specimens can acquire bony and soft tissue three-dimensional information using micro-CT, and even a single specimen allows cross-sectional observation in various planes^24–28^. The specimens were scanned using micro-CT with a 50-µm resolution (an image pixel size of 50 μm with 1024 × 1024 pixels). The scanned sequential images were re-sliced to be horizontal to the superior articular surface of the tibia using the ImageJ software. Then, using the “Radial slice” plugin of ImageJ software, the radial sequential images perpendicular to the articular surface were obtained. The radial slice plane was rotated clockwise at 1° intervals around the medial intercondylar tubercle in the posteromedial quadrant range (Supplementary Video S1).

### Statistical analyses

In six knees undergoing macroscopic analysis, statistical comparisons of the attachment widths of the joint capsule were performed using SPSS software version 27.0 (IBM Corp., Armonk, NY, USA). Distributions of all measurements consistently passed the Shapiro–Wilk test, and the data are expressed as mean and standard deviation. The comparisons of the attachment widths were analyzed using a one-way ANOVA. If the one-way ANOVA produced a significant result, the values for the regions were compared using post hoc Tukey's test. Both significance levels of the comparisons were set at *p* < 0.05. In addition, the effect size was calculated using G*Power software version 3.1.9.6, and post-hoc power analysis was performed. The effect size and power were 0.87 and > 0.99, respectively.

ICCs were also determined using a measurement process analysis. The qualitative cut-offs for ICCs are reported as follows: poor, < 0.40; fair, 0.40–0.59; good, 0.60–0.74; and excellent, 0.75–1.0^38^. All ICCs of the capsular attachment width were ≥ 0.89 (range, 0.89–1.000), indicating excellent agreement.

## Supplementary Information

Supplementary Figure S1.

Supplementary Video S1.

Supplementary Legends.

## Data Availability

The datasets used and/or analyzed during the current study are available from the corresponding author upon reasonable request.
