# Supplementary figures and images for: Posteromedial capsular anatomy of the tibia for consideration of the medial meniscal support structure using a multidimensional analysis
Source: Sci Rep. 2023 Jul 25;13:12030. doi: 10.1038/s41598-023-38994-x (PMC10368675; doi:10.1038/s41598-023-38994-x)

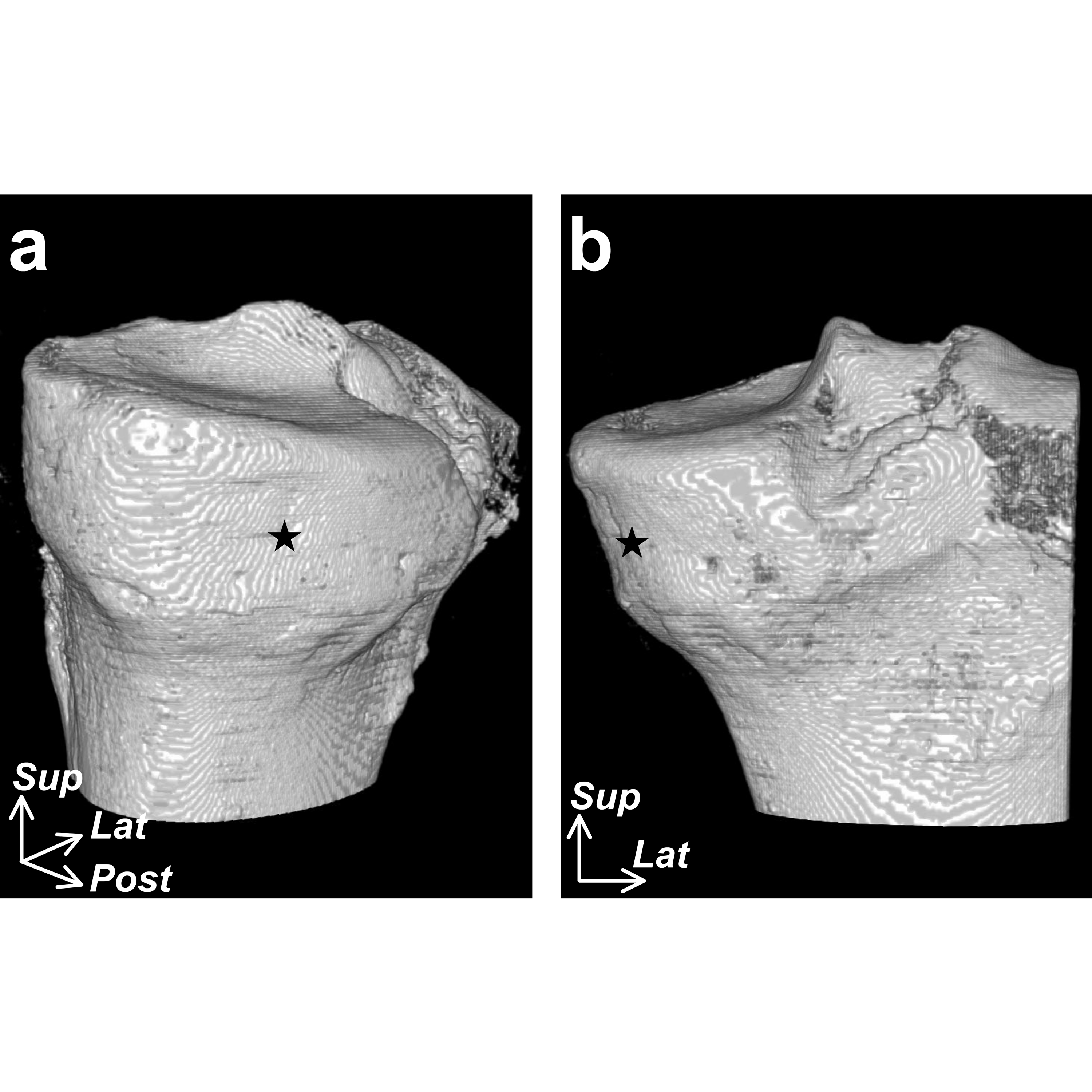

Supplement: Supplementary file 1 — Supplementary Figure S1. [file 41598_2023_38994_MOESM1_ESM.tif]
