# Supplementary material for: Posteromedial capsular anatomy of the tibia for consideration of the medial meniscal support structure using a multidimensional analysis
Source: Sci Rep. 2023 Jul 25;13:12030. doi: 10.1038/s41598-023-38994-x (PMC10368675; doi:10.1038/s41598-023-38994-x)
Supplement: Supplementary file 3 — Supplementary Legends. [file 41598_2023_38994_MOESM3_ESM.pdf]

Supplementary legends

Supplementary Figure S1. Bony configuration of the superior edge of the posteromedial tibia.

Micro-CT bony images on the posteromedial (**a**) and posterior (**b**) aspects. Star indicates the bony impression of the semimembranosus attachment.

Lat = lateral, Post = posterior and Sup = superior.

Supplementary Video S1. Serial radial sections movie of the joint capsule complex with the semimembranosus.

Left panel indicates the horizontal enhanced micro-CT image using phosphotungstic acid. Right panel indicates the radial slice images along the yellow line in the left panel, which centered around the medial intercondylar tubercle (MIT) and rotate clockwise in 1 degree-interval from sagittal sections. Scale bars = 5mm.

Cap = joint capsule, Gcm = medial head of the gastrocnemius, Gr = Gracilis, MM = medial meniscus, Po = popliteus, Sa = sartorius, Sm = semimembranosus, St = semitendinosus, Star = Sm attachment on the superior edge of the tibia, Ant = anterior, and Lat = lateral.
